# Chemotherapy-induced peripheral neuropathy—part 2: focus on the prevention of oxaliplatin-induced neurotoxicity

**DOI:** 10.1007/s43440-020-00106-1

**Published:** 2020-04-28

**Authors:** Kinga Sałat

**Affiliations:** Department of Pharmacodynamics, Jagiellonian University Medical College, 9 Medyczna St., 30-688 Kraków, Poland

**Keywords:** Oxaliplatin, Oxalate, Prevention strategies for CIPN, Duloxetine, Calmangafodipir, Clinical trials

## Abstract

**Background:**

Chemotherapy-induced peripheral neuropathy (CIPN) is regarded as one of the most common dose-limiting adverse effects of several chemotherapeutic agents, such as platinum derivatives (oxaliplatin and cisplatin), taxanes, vinca alkaloids and bortezomib. CIPN affects more than 60% of patients receiving anticancer therapy and although it is a nonfatal condition, it significantly worsens patients’ quality of life. The number of analgesic drugs used to relieve pain symptoms in CIPN is very limited and their efficacy in CIPN is significantly lower than that observed in other neuropathic pain types. Importantly, there are currently no recommended options for effective prevention of CIPN, and strong evidence for the utility and clinical efficacy of some previously tested preventive therapies is still limited.

**Methods:**

The present article is the second one in the two-part series of review articles focused on CIPN. It summarizes the most recent advances in the field of studies on CIPN caused by oxaliplatin, the third-generation platinum-based antitumor drug used to treat colorectal cancer. Pharmacological properties of oxaliplatin, genetic, molecular and clinical features of oxaliplatin-induced neuropathy are discussed.

**Results:**

Available therapies, as well as results from clinical trials assessing drug candidates for the prevention of oxaliplatin-induced neuropathy are summarized.

**Conclusion:**

Emerging novel chemical structures—potential future preventative pharmacotherapies for CIPN caused by oxaliplatin are reported.

**Graphical abstract:**

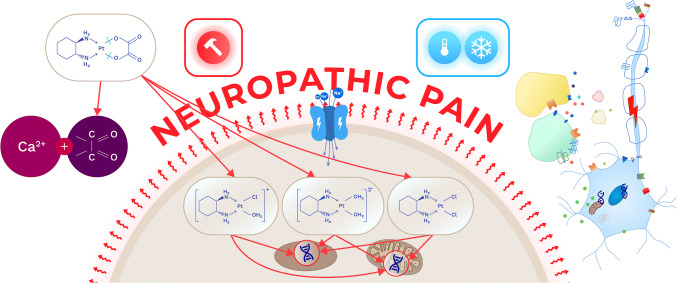

## Oxaliplatin as a neurotoxic drug

**Fig. 1:**
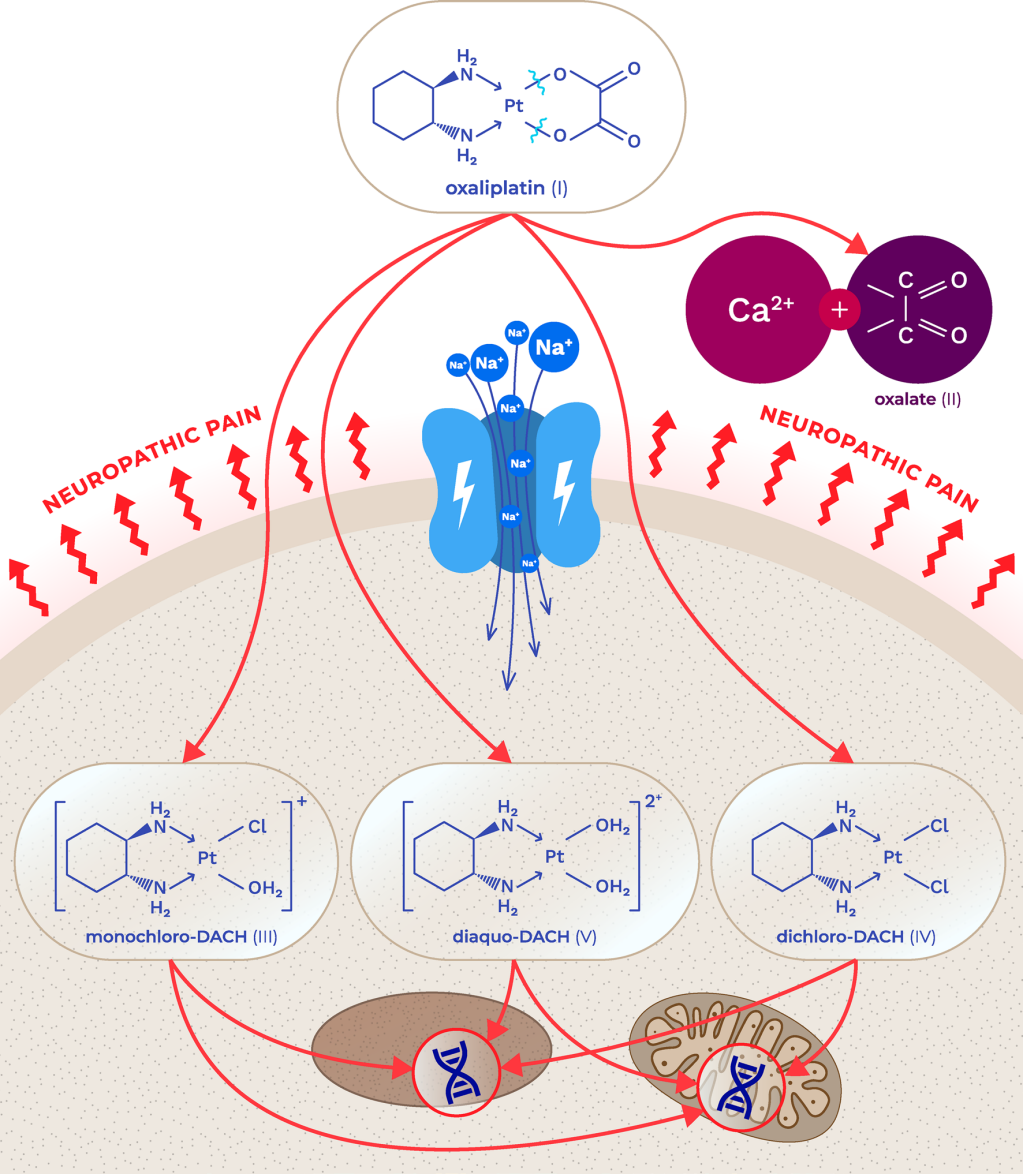
Chemical structure of oxaliplatin, its biotransformation pathways and a potential mechanism underlying the development of oxaliplatin-induced neuropathy: oxaliplatin (I) is rapidly hydrolyzed in vivo to bioactive derivatives through the displacement of the oxalate group by H_2_O and Cl^−^ ions to produce oxalate (II) as well as reactive monochloro-diaminocyclohexane (DACH) (III), dichloro-DACH (IV) and diaquo-DACH platinum (V) metabolites. Oxalate, which reacts with Ca^2+^ ions, is the main contributor to neurotoxicity caused by oxaliplatin

Oxaliplatin (*trans*-l-diaminocyclohexane oxalate platinum (II), Fig. 1), a third-generation platinum-based chemotherapeutic agent [1], has been placed on the World Health Organization’s List of Essential Medicines [2]. Similar to other platinum-based compounds (e.g., cisplatin, carboplatin), oxaliplatin interferes with tumor cell proliferation by forming deoxyribonucleic acid (DNA)-platinum adducts, which leads to cancer cell destruction [1].

Clinically, oxaliplatin is used in combination with folinic acid and 5-fluorouracil as a part of the FOLFOX regimen or with folinic acid, 5-fluorouracil and irinotecan (FOLFOXIRI) regimen as the first-line and as adjuvant colorectal cancer therapy [3].

Recent preclinical studies have shown that transporter-mediated uptake of oxaliplatin (and paclitaxel [4, 5]) into dorsal root ganglia neurons might trigger pathophysiological changes in sensory neurons, thus resulting in chemotherapy-induced peripheral neuropathy (CIPN) development [4].

The organic cation/carnitine transporters OCTN1 and OCTN2 belong to the organic cation transporter family. These proteins are responsible for transporting oxaliplatin and are regarded as key transporters maintaining the intracellular concentration of platinum derivatives via active uptake and efflux processes. OCTN1 and OCTN2 are also functionally expressed within dorsal root ganglia neurons, and OCTN1 is thought to be the main contributor to the neuronal accumulation of oxaliplatin and a mediator of its neurotoxicity [4, 6–8]. In addition, CTR1 (copper transporter 1), CTR2 (copper transporter 2), ATP7A (copper-transporting p-type adenosine triphosphatase 1) and ATP7B (copper-transporting p-type adenosine triphosphatase 2) are copper transporters that not only maintain homeostasis and copper metabolism but also are involved in cisplatin, carboplatin and oxaliplatin transport. A high CTR1 level is regarded as a predictor of prolonged survival and enhanced response to chemotherapy in cancer patients [9].

A unique feature of oxaliplatin is that it is rapidly and non-enzymatically transformed into several metabolites, including oxalate, monochloro-, dichloro- and diaquo-diaminocyclohexane (DACH) platinum metabolites (Fig. 1). This occurs via the replacement of the oxalate moiety with chloride ions and water in the blood plasma. The formation of these metabolites distinguishes oxaliplatin from cisplatin. Of note, oxalate is thought to be responsible for the cold-induced hypersensitivity observed in oxaliplatin-treated, but not cisplatin-treated, patients [10, 11], although additional mechanisms, including the binding of platinum complexes to cellular proteins, have also been suggested [12].

Oxalate is a well-known calcium chelator [13], and oxalate-induced pain is regarded as a consequence of the chelation of extracellular calcium ions (i.e., the removal of extracellular calcium ions), which in turn leads to the increase in sodium conductance, neuronal depolarization and hyperexcitability (Fig. 1) [14, 15].

An increase in extracellular calcium concentration increases the probability of voltage-gated sodium (Na_v_) channel closure and results in decreased excitability of peripheral neurons. Hence, the administration of calcium and magnesium ions before oxaliplatin infusion was evaluated in several clinical trials as a strategy to prevent the development of oxaliplatin-induced peripheral neuropathy, although clinical benefits of this approach were not confirmed [16, 17].

### Genetic polymorphisms associated with oxaliplatin-induced neuropathy

Data from mouse studies show that similarly to humans, various mouse strains exposed to oxaliplatin develop chemotherapy-induced peripheral neuropathy (CIPN). Significant and strain-dependent differences were observed in the severity and behavioral features of CIPN. This supports the hypothesis that, for oxaliplatin, a genetic component might be relevant for CIPN development, both in rodent models and in clinical settings. Importantly, these different neurotoxic phenotypes seem to be crucial in the choice of an appropriate mouse strain for CIPN studies [18].

Genetic risk factors associated with the development of CIPN have been studied recently [19]. As such, they can influence the absorption, distribution, metabolism or excretion of chemotherapeutic agents that are responsible for CIPN formation. It was suggested that polymorphisms of glutathione transferases, cytochrome P450 enzymes and ATP binding cassette transporters may be involved in the development of platinum-induced CIPN [5, 20–22]. The Ile105Val polymorphism of the *GSTP1* gene, encoding glutathione *S*-transferase P1, has been associated with a decreased risk of oxaliplatin-related CIPN. This mutation increases the activity of glutathione *S*-transferase P1, resulting in the increased conjugation of hydrophobic and electrophilic compounds with glutathione, thus reducing their toxicity [23].

In addition, genetic or pharmacological knockout of OCT2 prevented the development of cold and mechanical hypersensitivity following oxaliplatin therapy, suggesting that the OCT2 transporter plays a key role in oxaliplatin-induced cytotoxicity [24].

A polymorphism in the *ABCC2* (ATP binding cassette subfamily C member 2) gene coding for the multidrug resistance protein 2 (MRP2) that leads to higher oxaliplatin concentrations in neurons was associated with oxaliplatin-induced neurotoxicity [25]. Genetic variants of *AGXT* (alanine glyoxylate aminotransferase) gene coding for alanine glyoxylate aminotransferase (i.e., an enzyme involved in oxalate metabolism) have been correlated with the severity of CIPN in patients on oxaliplatin therapy [26]. The development of acute oxaliplatin-induced peripheral neuropathy was altered in patients with single-nucleotide polymorphisms in *SCNA* genes encoding selected Na_v_ channels. A polymorphism (rs2302237) in the *SCN4A* gene, which encodes the Na_v_1.4 channel and a polymorphism (rs1263292) in the *SCN10A* coding for Na_v_1.8 channel have been associated with an increased incidence of acute oxaliplatin-induced CIPN [27], while a polymorphism (rs6746030) in the *SCN9A* gene, which encodes the Na_v_1.7 channel, protected against severe oxaliplatin-induced CIPN [28].

### Clinical features of oxaliplatin-induced CIPN

In contrast to cisplatin, oxaliplatin is not nephrotoxic or ototoxic, but its use is still associated with various adverse effects, including myelotoxicity and CIPN. Neurotoxicity of oxaliplatin appears in two distinct forms, i.e., acute and chronic [29]. The acute, transient neuropathy develops in almost 90% of patients within hours of infusion and lasts a few days (usually 7 days), recurring with subsequent infusions. Acute neuropathy results from a transient impairment of ion channels and nerve hyperexcitability due to Na_v_ channel activation [30, 31] and is characterized by dysesthesia and paresthesia of the hands and feet. These symptoms are often exacerbated by cold. Motor symptoms (e.g., tetanic spasms, fasciculations, prolonged muscular contractions) can also be present in patients on oxaliplatin therapy. The acute form of oxaliplatin-induced CIPN tends to weaken between treatment cycles, but prolonged exposure to this drug often leads to the development of a severe chronic form of CIPN.

The incidence of chronic peripheral neuropathy following oxaliplatin treatment has been estimated as approximately 70% [32, 33]. Clinically, the symptoms of chronic oxaliplatin-induced neuropathy closely resemble those of the acute form and include paresthesia, hypoesthesia and dysesthesia of the hands and feet. Additionally, changed proprioception negatively affecting normal daily activities that require fine motor coordination might also occur [34, 35]. The main mechanism responsible for the observed permanent distal sensory loss is associated with the death of sensory neurons (i.e., nerve cell necrosis) due to the binding of oxaliplatin to mitochondrial DNA [36].

It has been estimated that the development of CIPN due to oxaliplatin administration is likely to occur at cumulative doses exceeding 780–850 mg/m^2^ [29, 35, 37, 38]. The symptoms that occur may worsen or appear weeks to several months after the completion of therapy (‘coasting’ phenomenon), and the estimated prevalence of these persistent symptoms is approximately 60% of patients; these patients report long-lasting neuropathic symptoms that negatively interfere with their daily functions and significantly impair their quality of life [29, 34]. Importantly, overall recovery from CIPN symptoms is usually incomplete and might last up to five years following oxaliplatin chemotherapy cessation [39].

Symptoms of acute and chronic neuropathy were evaluated in patients receiving an adjuvant FOLFOX regimen in a phase III clinical trial (ClinicalTrials.gov: NCT00316914). Acute neuropathy was assessed for 6 days with each cycle of FOLFOX. Chronic neuropathy was evaluated until 18 months after chemotherapy cessation. Acute neuropathy symptoms included sensitivity to touching cold items, sensitivity to swallowing cold items, throat discomfort, and muscle cramps. Acute symptoms peaked at day 3 and improved, but they tended not to resolve completely between treatments. For chronic neurotoxicity, tingling was the most severe symptom, followed by numbness and then pain. Patients with more severe acute neuropathy during the first cycle of therapy experienced more severe chronic sensory neurotoxicity [40].

## Available preventative therapies for CIPN caused by oxaliplatin

Considering the severity of CIPN and its irreversibility in a large proportion of patients, for many years efforts have been being made to alleviate this pharmacoresistant clinical condition. Therefore, the National Cancer Institute's Symptom Management and Health-Related Quality of Life Steering Committee has announced that CIPN is a priority area of translational research in cancer care [4]. Even the latest literature data clearly indicate that although numerous preventative therapies were tested for their potential utility for CIPN alleviation, this clinical entity currently is still not preventable [41–43], and many strategies tested were found to be ineffective (Table 1). Therefore, based on meta-analyses of clinical trials, at present, no drug can be proposed as a gold standard to either prevent CIPN or treat its symptoms, and chemotherapeutic drug dose modification remains the only preventative strategy [41].

**Table 1:** A summary of ASCO recommendations for preventative therapies for CIPN [41, 44–50]

Strength of recommendation	Drug
Recommendation strong against	Acetyl-l-carnitine, diethyldithiocarbamate (DDTC), nimodipine, BIA 10-2474
Recommendation moderate against	Amifostine, amitriptyline, calcium and magnesium infusions, glutathione, Org-2766 (ACTH analog), pregabalin, retinoic acid, emfilermin (rhuLIF), vitamin E
Inconclusive data: balance of benefits and harms	Acetylcysteine, alpha-lipoic acid, carbamazepine, oxcarbazepine, glutamate, glutamine, goshajinkigan, neurotropin, omega-3 fatty acids, venlafaxine, vitamin B complex
Recommendation moderate for	Duloxetine
Recommendation strong for	None

Several clinical trials aimed to examine potentially neuroprotective therapies to prevent CIPN. Based on the data obtained, only duloxetine is moderately recommended by the American Society of Clinical Oncology (ASCO) for the prevention of oxaliplatin-induced CIPN (Table 1) [44, 45]. These guidelines undoubtedly show that there are no clear algorithms for CIPN prevention because high-quality and strong evidence for the preventative action of agents tested is not available.

It should also be noted that many other drugs have been assessed, but their utility for the prevention of oxaliplatin-induced CIPN is still controversial (Table 1). For example, venlafaxine and neurotropin are also not recommended for routine use because of insufficient information on their clinical efficacy. A pilot randomized, placebo-controlled, double-blinded phase III trial (ClinicalTrials.gov: NCT01611155) tested the use of venlafaxine to prevent oxaliplatin neurotoxicity in 50 patients with stage II-IV colon cancer. The results of this study supported neither the use of venlafaxine for the prevention of oxaliplatin-induced neuropathy in clinical practice nor the initiation of a further phase III trial for an investigation of venlafaxine in this setting [51].

Pregabalin was also assessed in a phase III, randomized, double-blind, placebo-controlled clinical trial for its efficacy and safety in the prevention and reduction of oxaliplatin-induced painful neuropathy (PreOx; ClinicalTrials.gov: NCT01450163). Patients received either pregabalin or placebo for 3 days before and 3 days after each oxaliplatin infusion and were followed for up to 6 months. The preemptive use of pregabalin during oxaliplatin infusions was safe, but it did not decrease the incidence of CIPN (Table 1) [41, 52].

Carbamazepine, oxcarbazepine, glutamate and glutamine, goshajinkigan, oral alpha-lipoic acid, omega-3 fatty acids and oral vitamin B complex are not recommended as CIPN-preventing drugs because of their inconclusive data, low level of evidence and low strength of available evidence (Table 1) [41, 45, 48, 50, 53–57].

Other trials on potentially promising drug candidates for CIPN prevention, such as a fatty acid amide hydrolase inhibitor (BIA 10-2474, Table 1), were prematurely terminated due to serious adverse events noted in participants (death, severe neurological complications) [44].

### Duloxetine

Evidence from preclinical [58, 59] and clinical studies [4] suggests that duloxetine, an antidepressant drug acting as a serotonin-noradrenaline reuptake inhibitor (SNRI), may be effective in the prevention of CIPN without reducing the antitumor activity of oxaliplatin [49]. As shown in Table 1, ASCO recommends duloxetine as the only potential treatment for CIPN, but importantly, this guidance still lacks the support of sufficient evidence. Moreover, since in clinical trials duloxetine was not fully effective and it did not work in every patient enrolled, the identification of strong predictors for duloxetine response is a priority area of research. Results from a phase III double-blind trial of oral duloxetine for the treatment of pain associated with chemotherapy-induced peripheral neuropathy (ClinicalTrials.gov: NCT00489411) revealed that oxaliplatin-treated patients with high emotional functioning are more likely to experience pain reduction resulting from duloxetine administration [14].

Since patients with oxaliplatin-induced painful CIPN are more likely to benefit from the use of duloxetine than those with paclitaxel-induced CIPN, this also suggests that duloxetine’s mechanism of action may be closely associated with very specific molecular mechanisms underlying oxaliplatin-induced neurotoxicity [14, 60]. This possibility has not been fully explained, so far, and it seems to be even more exciting in terms of a distinct clinical efficacy of duloxetine and venlafaxine, both being representatives of SNRI antidepressants. Their efficacy was directly compared in cancer patients who were suffering from CIPN. The administration of duloxetine was more effective than that of venlafaxine in decreasing the symptoms of peripheral neuropathy due to chemotherapy. Duloxetine had a better effect on reducing the grade of motor neuropathy and neuropathic pain severity [61]. A potential explanation for this observed diversity in effectiveness is the difference in the pharmacological profile of both drugs. Compared to other SNRIs, venlafaxine has a high affinity for the serotonin transporter (SERT) but a relatively lower affinity for the norepinephrine transporter (NET), so at lower doses, it may act as a selective serotonin reuptake inhibitor (SSRI) and may not have the same activity against painful neuropathy as duloxetine [51].

## Drug candidates for the prevention of CIPN caused by oxaliplatin

**Fig. 2:**
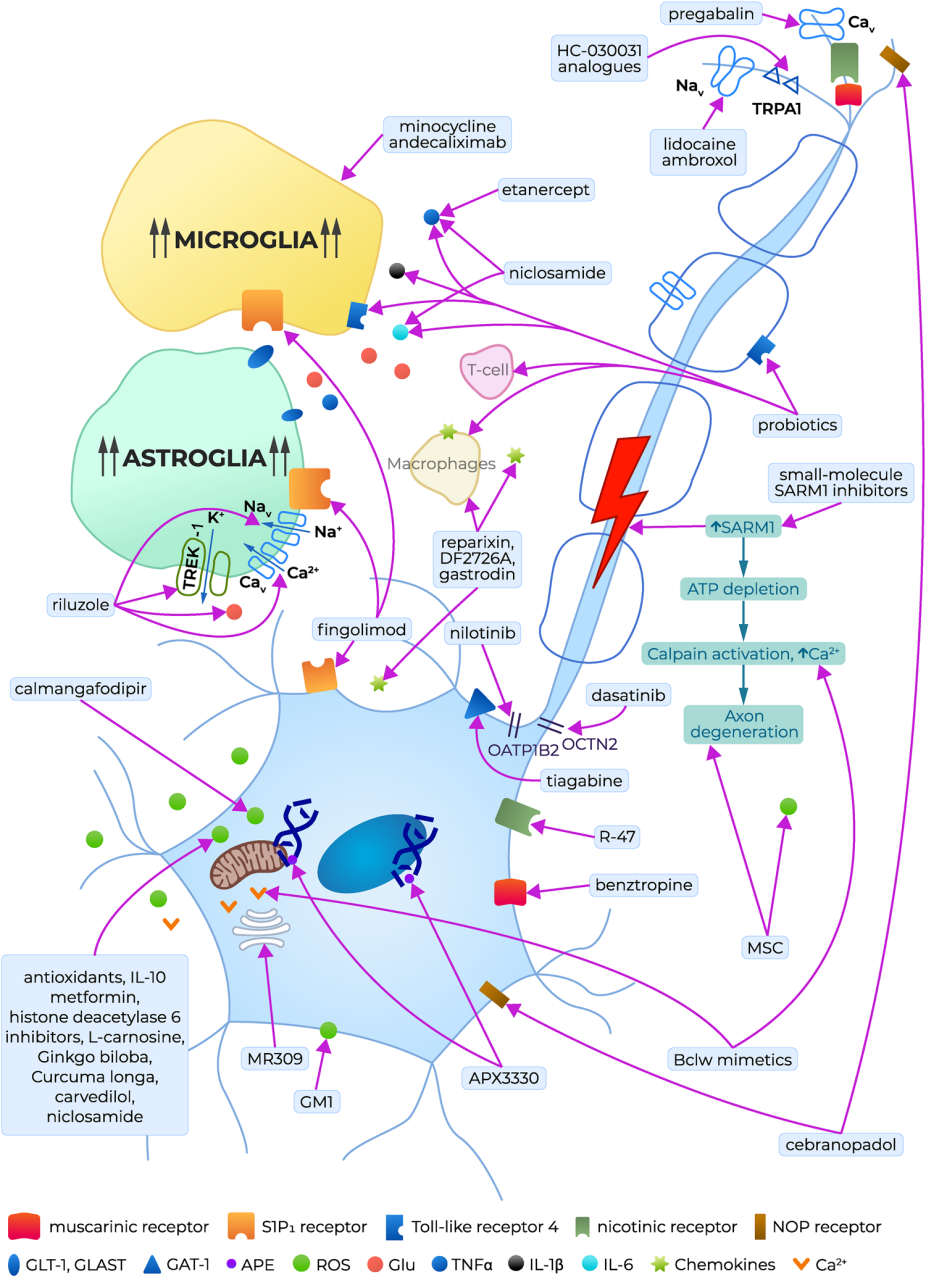
Potential preventative therapies for CIPN caused by oxaliplatin: clinically tested drug candidates, repurposed drugs and preclinically tested lead compounds. *APE* apyrimidinic endonuclease/redox effector factor, *GAT-1* γ-aminobutyric acid (GABA) transporter isoform 1, *Glu* glutamate, *GLT-1* glutamate transporter 1, *GLAST* GLutamate and ASpartate Transporter, *GM1* monosialotetrahexosylganglioside, *ROS* reactive oxygen species, *TLR4* toll-like receptor 4, *Na*_*v*_ voltage-gated sodium channels, *Ca*_*v*_ voltage-gated calcium channels, *TREK-1* two-pore-domain background potassium channel, *TRPA1* Transient Receptor Potential Ankyrin-repeat 1 channel, *IL* interleukin, *TNFα* tumor necrosis factor α, *SARM-1* sterile alpha and TIR motif-containing protein 1, *ATP* adenosine triphosphate, *Bclw* Bcl2 family member, *NOP* nociceptin opioid peptide receptor, *S1P*_*1*_ sphingosine-1-phosphate receptor type 1, *OATP1B2* solute carrier organic anion-transporting polypeptide B2, *OCTN2* Na^+^-dependent organic cation/carnitine transporter, *MSC* mesenchynmal stem cell therapy

Since CIPN is not preventable using currently available drugs, there is a strong medical demand to search for novel therapeutic options for this clinical entity. Our understanding of the complexity of neurobiological mechanisms implicated in CIPN symptomatology (altered ion channel expression, neuronal hyperexcitability, mitochondrial dysfunction, impaired immune cell signaling and axon degeneration) has constituted a basis for extended research on pharmacological prevention of CIPN. In this context, not only novel chemical compounds but also repurposed drugs are being investigated at preclinical and clinical stages of research. Considering the risk factors and causes of CIPN development, several potential approaches have been proposed (Fig. 2) [44].

An ‘ideal’ CIPN-preventing drug should have the following features: (i) it should act as a multitarget agent, as this might increase its protective efficacy; (ii) it should not diminish the antitumor efficacy of the chemotherapeutic used; and (iii) it should be introduced into therapy relying on an individual patient’s reported outcomes to increase its efficacy in preventing the symptoms and causes of CIPN. Several experimental therapies for CIPN prevention have reached the clinical stage of testing. These emerging drug candidates are shown in Table 2 and Fig. 2.

**Table 2:** Emerging drug candidates tested in clinical trials for CIPN prevention [4, 62]

Agent	Mechanism of action/target	Patient population	Clinical trial number
Nilotinib (oral, at the recommended phase II dose, which will be established as a dose that significantly inhibits OATP1B1 without causing changes in the pharmacokinetic profiles of paclitaxel)	Tyrosine kinase inhibitor, OATP1B2 transporter inhibitor [5]	Stage I–III breast cancer patients initiating paclitaxel therapy	NCT04205903 (phase Ib, II)^b^
Dasatinib (oral, at the recommended phase II dose, which will be defined as the lowest intermittent dose of dasatinib that affects serum biomarkers of OCTN2 without influencing the pharmacokinetic properties of oxaliplatin)	OCTN2 transporter inhibitor [63]	Stage IV colorectal cancer patients initiating FOLFOX and bevacizumab	NCT04164069 (phase Ib)^c^
Calmangafodipir (PledOx; intravenous: 2, 5, 10 µmol/kg) [64]	Reactive oxygen species reduction	Stage IV colorectal cancer patients initiating oxaliplatin therapy (POLAR M, POLAR A)	Polar M: NCT03654729 (phase III)^c^ POLAR A: NCT01619423 (phase II)^d^ NCT04034355 (phase III)^c^, SUNCIST NCT03430999 (phase I)^d^
APX3330 (oral, 60, 120 mg twice daily)	Increase in APE1 expression	Patients with advanced solid tumors	NCT03375086 (phase I)^c^
Fingolimod (FTY-720; oral, once daily; dose regimen not available)	S1PR1 antagonist [65, 66]	Breast cancer patients initiating adjuvant paclitaxel therapy	NCT03941743 (phase I)^c^
GM1 (monosialotetrahexosylganglioside; intravenous 80, 120 mg/day) [67]	Lipid peroxidation inhibitor	Patients with colorectal cancer initiating oxaliplatin adjuvant therapy	NCT02251977 (phase III)^d^
l-Carnosine (oral, 500 mg/day) [68]	Lipid peroxidation inhibitor, reactive oxygen species reduction, anti-inflammatory, anti-apoptotic [69]	Colorectal cancer patients initiating oxaliplatin therapy	NCT02808624 (phase I)^d^
A polyamine-reduced diet^a^ (PRD) [37]	Decrease in NMDA receptor activity; reduction of pain chronification	Patients initiating oxaliplatin therapy (gastrointestinal cancer, without cytotoxic neurotoxic chemotherapy) had to receive FOLFOX4 in the adjuvant, neoadjuvant or palliative situation with an expected period of 8 rounds of treatment or 4 months	NEUROXAPOL (NCT01775449) (phase III)^d^
Riluzole (oral, 50 mg tablet/twice a day) [70]	Neuroprotectant by preventing excessive glutamate accumulation in the nervous system [70]; TREK-1, TRAAK potassium channel opener [71]	Patients ≥ 18 years old that have developed stage II/III colorectal cancer and are eligible for simplified FOLFOX4 (6–12 cycles) adjuvant chemotherapy	NCT03722680 (phase II) ^b^
Lidocaine [intravenous, 1 mg/kg infusion (based on ideal body weight (IBW)] over 10 min, followed by a 0.04 mg/kg/min infusion over an additional 120 min, resulting in a total dose of 5.8 mg/kg IBW) [72]	Na_v_ channel inhibitor [73]	Stage III and IV colorectal cancer Scheduled for oxaliplatin treatment in mFOLFOX6-based chemotherapy	NCT03254394 (phase I, phase II)^c^
Metformin (oral, 1000 mg/twice a day for 12 days) [73]	Indirect AMP-activated protein kinase activator [74]	Breast cancer patients initiating paclitaxel therapy	NCT02360059 (phase II—terminated due to low accrual)
MR309 (a.k.a. E-52862, oral, 400 mg/day, single daily administration, 5 days per cycle and starting the day before the cycle, up to a maximum of 12 cycles of oxaliplatin) [75, 76]	Sigma-1 receptor antagonist [76, 77]	Patients ≥ 18 years old that have developed stage II-IV colorectal cancer Scheduled for oxaliplatin treatment in FOLFOX4 or FOLFOX6-based chemotherapy	EudraCT number 2012-000398-21 (phase II)^d^
EMA401 (a.k.a. olodanrigan, oral, 100 mg twice daily for 28 days) [78]	Angiotensin II type 2 receptor antagonist [79–82]	Patients ≥ 18 years old with CIPN symptoms caused by taxanes or platinum chemotherapy used for any cancer type	EudraCT number 2011-004033-13 (phase II)^d^

^a^Containing less than 10 mg/kg of polyamines (putrescine, spermidine and spermine)

^b^Study not yet recruiting

^c^Study recruiting patients now

^d^Study completed

### Targeting chemotherapeutic drug uptake transporters: tyrosine kinase inhibitors

In vitro and in vivo studies have shown that two tyrosine kinase inhibitors, nilotinib (an OATP1B2 inhibitor) and dasatinib (an OCTN2 inhibitor), might offer a potential neuroprotective strategy for CIPN caused by paclitaxel and oxaliplatin (Fig. 2) without negatively impacting their systemic clearance or antitumor efficacy. As repurposed drugs, nilotinib and dasatinib are currently being tested in an ongoing phase Ib trial and in double-blinded, placebo-controlled, randomized phase II studies (Table 2) [4].

### Targeting mitochondrial dysfunction and oxidative stress

Oxidative stress caused by chemotherapy is a significant contributor to mitochondrial damage, and reactive oxygen species (ROS) neutralization is a potential preventative strategy for CIPN. Manganese superoxide dismutase (MnSOD) catalyzes the dismutation of superoxide and forms hydrogen peroxide and molecular oxygen. Hence, this enzyme limits the reaction of superoxide with nitric oxide, thus preventing the formation of the reactive and highly toxic nitrogen species peroxynitrite, which is able to nitrate the tyrosine residue of tissue proteins [64]. Recently, targeting MnSOD has emerged as a very promising strategy to prevent CIPN caused by oxaliplatin [83–86].

#### Calmangafodipir

Calmangafodipir (Ca4Mn(DPDP)_5_, PledOxVR), a derivative of mangafodipir, a magnetic resonance imaging contrast and a cytoprotectant agent [87, 88], is a mitochondrial MnSOD mimetic that reduces ROS tissue levels (Fig. 2). Although preclinical and clinical data have confirmed that such compounds have significant preventative neuroprotective activity in oxaliplatin-induced CIPN [83–86], it is hypothesized that neuroprotection from calmangafodipir may also be possible with other chemotherapeutics [4, 64].

In a placebo-controlled, double-blinded, randomized, phase II study in patients with metastatic colorectal cancer (Table 2), calmangafodipir reduced cold allodynia and other sensory symptoms during and after treatment with oxaliplatin. The neuroprotective effects seen were clinically meaningful, as a delay in both the onset and the reduction of the intensity of CIPN symptoms were noted both in the acute and in the chronic phase of neuropathy. In this trial, progression-free and overall survival outcomes were not negatively influenced by calmangafodipir [64].

Two international trials, POLAR A and POLAR M are currently evaluating the efficacy of calmangafodipir for the prevention of oxaliplatin-induced neuropathy in colorectal cancer patients. Results are expected in the years 2020/2021 (POLAR A) or 2021–2023 (POLAR M) [4, 62].

Apart from CIPN, calmangafodipir has also shown efficacy in severe paracetamol overdose and, based on results from a clinical trial (POP Trial, Clinicaltrials.gov: NCT03177395), might be regarded as an adjuvant or alternative to the *N*-acetylcysteine antidote for paracetamol overdose [89, 90]. In this trial, the most frequent adverse effect was gastrointestinal toxicity (nausea, vomiting, diarrhea, dyspepsia) [90–92].

### Targeting apurinic/apyrimidinic endonuclease function

Another mechanism of nerve injury that underlies CIPN formation is associated with impaired DNA repair within the sensory nervous system. The pathway for base excision repair is the primary means for the repair of oxidative DNA damage. In this pathway, the enzyme apyrimidinic endonuclease/redox effector factor (APE1) is an important molecule for the removal of the damaged bases. Decreased APE1 levels in sensory neurons increase neurotoxicity and targeting APE1 by the small molecules APX3330 and APX2009 (Fig. 2) provided neuroprotection against cisplatin- and oxaliplatin-induced neurotoxicity. APX2009 also demonstrated a strong tumor cell-killing effect. These data suggest that such compounds might be effective in preventing or reversing platinum-induced CIPN without reducing the anticancer activity of platinum-based chemotherapeutics [93]. Currently, APX3330 is entering clinical trials as an antineoplastic agent as well for the prevention of CIPN (Table 2) [94].

### Targeting the inhibition of neuronal apoptosis and astrocyte activation

Impaired sphingolipid metabolism is also considered a mechanism contributing to CIPN development. Certain chemotherapeutics, including paclitaxel [66], oxaliplatin [95] and bortezomib [96], induce the development of CIPN by dysregulating sphingolipid metabolism, leading to increased formation of sphingosine-1-phosphate (S1P), which binds and stimulates sphingosine-1-phosphate receptor 1 (S1PR1). In rodent models of CIPN, the ceramide metabolic pathway was activated in the spinal cord, and blocking the formation of S1P with sphingosine kinase inhibitors reversed CIPN symptoms, mainly allodynia and hyperalgesia [66].

Fingolimod (FTY720; Table 2, Fig. 2) acts as a nonselective agonist of sphingosine-1-phosphate receptors (S1P1R, S1P3R, S1P4R, and S1P5R but not S1P2R). Fingolimod phosphate activates S1P1Rs with high potency (*K*_D_ = 0.3 nM) and with higher efficacy than S1P. The overactivation of S1P1R caused by fingolimod results in rapid receptor desensitization and internalization. Thus, fingolimod behaves as a selective functional antagonist of the S1P1R subtype through the induction of receptor downregulation. The downstream effect of S1P1R blockade is the inhibition of the NF-κB pathway [97]. At present, fingolimod is commercially available as a marketed drug for multiple sclerosis, but its daily injections also inhibited the development of mechanical allodynia and hyperalgesia induced by paclitaxel, oxaliplatin, and bortezomib. Similar effects were also noted with other S1P1R antagonists. Moreover, a prolonged treatment with fingolimod or other S1P1R functional antagonists did not induce tolerance to their analgesic effects. S1P1R antagonists inhibited the development of CIPN, but they were also able to induce a sustained reversal of paclitaxel-induced neuropathic pain. Apart from fingolimod, novel S1PR modulators are being developed, including ponesimod [66], siponimod [98], and CYM5442, as well as selective S1P1R antagonists, including W146, NIBR-14/15, and TASP0251078, as novel drug candidates for the treatment of various diseases, including multiple sclerosis, rheumatoid arthritis, colitis and cancer [99–101]. Importantly, the S1PR1 antagonists developed to date are not expected to reduce the anticancer activity of chemotherapeutic agents, and they may act synergistically [97, 102], making fingolimod and its analogs attractive agents for CIPN prevention [101]. Transient cardiovascular adverse effects of fingolimod result from a transient activation of S1P1R—a phenomenon that precedes its functional antagonism. These cardiovascular adverse effects include first-dose bradycardia, atrioventricular conduction block and hypotension, and they must be cautiously considered when utilizing this drug for CIPN prevention and treatment [4].

Monosialotetrahexosylganglioside (GM1) is a glycosphingolipid located in the outer layer of the plasma membrane (Fig. 2). It is critical for nerve development, differentiation and repair after the injury as it has neuroprotective, neurotrophic-factor-like activity by activating the Trk neurotrophin receptors [103, 104]. GM1 can also prevent seizures and oxidative stress [105]. Initially, GM1 was approved for the treatment of vasculogenic or traumatic central nervous impairments and Parkinson’s disease, and now its potential procognitive activity is also suggested [103].

In a clinical trial conducted in colon cancer patients on FOLFOX therapy (Table 2), GM1 attenuated symptoms of acute neuropathy (cold sensitivity, throat discomfort, muscle cramps), but its use to prevent the neurotoxicity of oxaliplatin is currently not recommended, and additional placebo-controlled studies are needed to better assess the utility of this agent in this clinical condition [4, 67].

### Sodium channel inhibition

Na_v_ channels are an important target for analgesic drugs used in neuropathic pain conditions. Most of the available Na_v_ inhibitors nonselectively block Na_v_ channels within the sensory nervous system. From these drugs, lidocaine (Table 2, Fig. 2), a local anesthetic agent, will be assessed in a pilot study to determine the tolerability and the efficacy of its intravenous administration for the prevention of oxaliplatin-induced cold hypersensitivity and spontaneous pain in the setting of mFOLFOX6 chemotherapy for advanced colorectal cancer [72].

Among Na_v_ channels, Na_v_1.7 plays an important role in multiple neuropathic pain states. It was shown that the expression and function of Na_v_1.7 are increased in preclinical models of CIPN [106, 107]. Data from a randomized, double-blind, placebo-controlled pilot study investigating the efficacy of a Na_v_1.7 antagonist, XEN402, indicate that it is effective in patients with inherited erythromelalgia, but so far, this compound has not been assessed in CIPN [4].

### Sigma-1 receptor antagonism

Selective sigma-1 receptor antagonists (e.g., MR309) attenuated oxaliplatin-induced CIPN [76], and it was suggested that sigma-1 receptors might constitute a novel drug target for CIPN-preventing drugs [77]. Currently, MR309 (400 mg/day, 5 days per cycle) has completed a phase II randomized, double-blind and placebo-controlled clinical trial in patients with colorectal cancer receiving FOLFOX, showing efficacy and a good safety profile in patients on oxaliplatin therapy [76]. Compared to placebo, MR309 significantly reduced the cold pain threshold temperature and lowered the proportion of patients with severe chronic neuropathy. MR309 increased the total amount of oxaliplatin delivered and it attenuated motor hyperexcitability symptoms in patients with colorectal cancer treated with oxaliplatin. A potential neuroprotective role of MR309 for acute and chronic CIPN induced by oxaliplatin was demonstrated. Taken together, it has been suggested that MR309 might be a first-in-class drug with a novel mechanism of action to improve some signs and symptoms of acute CIPN (e.g., cold pain and motor symptoms) caused by oxaliplatin. However, it has to be noted that further studies should be focused on testing different dose regimens of MR309 administration and continuous dosing during the full chemotherapy period [76].

### Angiotensin II type 2 receptor antagonism

EMA401 (olodanrigan) is an angiotensin II type 2 (AT_2_) receptor antagonist. In clinical trials, this orally active agent was tested in patients with postherpetic neuralgia, painful diabetic neuropathy [79–82] and CIPN [78]. Although EMA401 showed antineuropathic properties in the postherpetic neuralgia and painful diabetic neuropathy patient populations, these trials were withdrawn or terminated due to the side effects observed [108–111].

A phase II clinical trial of EMA401 in patients suffering from CIPN was conducted as an open-label biomarker study designed to provide a proof of concept of the use of EMA401 in CIPN due to taxanes or platinum derivatives. In this trial, the primary endpoint was the change (i.e., a decrease of at least 30%) in mean spontaneous pain intensity score between baseline and the last week of 28 days of EMA401 dosing. A number of secondary endpoints were also evaluated, including changes in nerve characteristics in skin biopsies taken from the calf, change in evoked (light touch) mean pain intensity score from baseline over time, change in evoked (cold touch) mean pain intensity score over time and patient global impression of change [109]. It has to be noted that the results of this trial are inconclusive and difficult for interpretation because statistical analyses have not been specified as only one arm was reported [78].

## Future outlook: data from preclinical studies

Although the mechanisms underlying CIPN are relatively well understood, the available results of clinical trials clearly indicate that the success rate for the introduction of novel drugs that are able to prevent CIPN is low [50], the progress in this area is slow and, at present, there is insufficient evidence to recommend any specific agent for CIPN prevention. Hence, animal research is a strong scientific and practical demand, because it enables the discovery and development of novel interventions for this clinical entity. Since mechanism-based therapies are urgently needed [112], novel targets are being explored to develop potential drug candidates. However, the drug development process is both time- and cost-consuming. Therefore, drug repurposing, i.e., the identification of new clinical applications for registered drugs, is also a valuable alternative approach to drug discovery and development research [113].

In this context, targeting sterile alpha and TIR motif-containing protein 1 (SARM-1), an injury-inducible nicotinamide adenine dinucleotide glycohydrolase (NADase) that triggers axon loss, is an emerging strategy for the prevention of CIPN-related axon degeneration (Fig. 2), and some SARM-1-targeting agents are currently in development. In addition to small-molecule inhibitors, a very potent, dominant-negative version of SARM-1 was developed. It acts by blocking the activation of wild-type SARM-1, and this protects axons from damage. Gene therapy using AAV-mediated delivery of this SARM-1 dominant-negative molecule provided long-lasting axonal protection [114]. In addition, SARM1-mediated NADH dehydrogenase subunit I (NAD1) destruction and loss is compensated by the development of NAD1 precursors, which have the potential ability to protect axons. These NAD1 precursors are natural products that have potential utility in CIPN prevention [114]. In addition to the direct influence on SARM-1, targeting the upstream pathways regulating SARM-1 to prevent CIPN is of interest. Efforts have been made to boost the function and increase the expression of nicotinamide nucleotide adenylyltransferase 2 (NMNAT2). Targeting the degradation of NMNAT2 might also be an alternative for neuropathy [114–116].

Ethoxyquin was recently identified as a neuroprotective compound showing potential benefits in toxic neuropathies. Its efficacy without compromising antitumor efficacy was demonstrated against paclitaxel-induced CIPN in vivo. Ethoxyquin also prevented cisplatin-induced neurotoxicity in vitro. In vivo, chronic administration of ethoxyquin partially attenuated cisplatin-induced abnormalities. The neuroprotective effect of ethoxyquin is thought to result from the inhibition of the chaperone activity of heat shock protein, Hsp90 [117, 118].

Bclw is a death-protecting member of the Bcl2 family of apoptosis-regulating proteins. The increased levels or activity of Bclw might also represent a novel therapeutic target for the prevention of CIPN (Fig. 2). Bclw was shown to play a role in paclitaxel-induced CIPN. Paclitaxel reduced axonal Bclw synthesis, altered intracellular calcium flux and activated calpain proteases. It also selectively impaired axonal trafficking of RNA granules and initiated inositol trisphosphate (IP_3_)-receptor-dependent axon degeneration [119]. Thus, the modulation of Bclw levels [101], as well as the design of Bclw mimetics [119], might represent another novel therapeutic strategy for CIPN prevention.

Since oxidative stress in the nervous system is one of triggering factors for CIPN, and an impaired mitochondrial antioxidant response after paclitaxel, oxaliplatin and bortezomib is thought to underlie CIPN [120], novel mitochondria-targeting antioxidants have also been evaluated. In mice, SS-31 prevented symptoms of acute neuropathy caused by oxaliplatin [121].

In CIPN, for the protection of mitochondria, the inhibition of nitro-oxidative stress with novel potential drugs might also include the use of the mitochondrial protectant pifithrin-µ, which acts by inhibiting mitochondrial p53 accumulation [122, 123], histone deacetylase 6 inhibitors [124], metformin [44, 74, 125], peroxynitrite decomposition catalysts [126], and antioxidants and anti-inflammatory cytokines, such as interleukin (IL) 10 [125, 127] (Fig. 2).

In addition, the targeting of inflammation and its markers in glial cells and peripheral immune system might contribute to CIPN prevention. Etanercept, a tumor necrosis factor-α (TNFα)-targeting antibody, decreased levels of IL-1α, IL-1β, IL-6, TNFα, interferon γ (INF-γ) and monocyte chemoattractant protein-1 (MCP-1), (Fig. 2) and reduced painful symptoms caused by paclitaxel, showing a potential utility of etanercept as a CIPN-preventing drug [128].

The neuroprotective potential of niclosamide in CIPN induced by oxaliplatin was confirmed in mice, normal neuron-like cells and cancer cells. In neuron-like cells, niclosamide reduced the production of oxaliplatin-mediated hydrogenium peroxide, thereby preventing cell death. In colon cancer cells, niclosamide enhanced oxaliplatin-mediated cell death through increased hydrogenium peroxide production. In neuropathic mice, niclosamide prevented tactile hypoesthesia, thermal hyperalgesia and decreased neuronal hyperexcitability. Niclosamide prevented oxaliplatin-induced increased levels of IL-6 and TNFα (Fig. 2) and reduced oxidative stress. Taken together, niclosamide displayed antitumor and neuroprotective effects without reducing the antitumor efficacy of oxaliplatin [129].

As mentioned above, peripheral immune system cells such as macrophages and monocytes have been shown to be targets of CIPN mechanisms. In this context, another potential strategy to prevent CIPN is the targeting of chemokines and their receptors. For instance, blocking MCP-1 (CCR2) signaling might be an effective therapeutic strategy to prevent or reverse CIPN caused by paclitaxel [130].

Chemokine receptor CXCR1/CXCR2 ligands also show some promising CIPN-preventing properties. Brandolini and colleagues investigated the effect of reparixin, an inhibitor of CXCR1/CXCR2, in the suppression of the development of paclitaxel-induced neuropathic pain in rats. Reparixin attenuated paclitaxel-induced mechanical and cold allodynia in rats. This study suggested that the inhibition of CXCR1/CXCR2 combined with standard taxane therapy, in addition to potentiating the taxane antitumor activity, can reduce paclitaxel-induced neurotoxicity, thus giving some insight for the development of novel treatments [131]. Dual inhibitors of CXCR1/CXCR2 were also tested in the oxaliplatin-induced neuropathic pain model. One of such compounds, DF2726A, reduced pain behavior in oxaliplatin-treated rats. This study confirmed that IL-8 (CXCL8) and its receptors CXCR1/CXCR2 [132] are involved in the development of CIPN caused by oxaliplatin, and blocking the CXCL8 pathway by DF2726A can significantly counteract neurotoxic effects resulting from oxaliplatin administration [133].

Novel therapies based on chemokine CX3CL1 (fractalkine) and its receptor expressed on monocytes, [134] have also emerged as potential options to alleviate CIPN symptoms [135]. It has been shown that gastrodin relieved vincristine-induced CIPN in rats by inhibiting the activation of spinal microglia through CX3CL1 and its receptor, CX3CR1 [136].

Using the oxaliplatin model of CIPN, it has also been demonstrated that the increased expression of C–C chemokine ligand 2 (CCL2) and its receptor, CCR2, in the dorsal root ganglia was lowered after intrathecal administration of anti-CCL2 antibodies, which prevented the development of oxaliplatin-induced mechanical hypersensitivity [137].

In a mouse model of oxaliplatin-induced CIPN, it has also been demonstrated that thrombomodulin α, a recombinant human soluble thrombomodulin, prevented allodynia, most likely via a thrombin-dependent antineuropathic action. The oral anticoagulants warfarin, dabigatran and rivaroxaban suppressed this beneficial antineuropathic effect of thrombomodulin α [138].

Matrix metalloproteinases (MMPs) are also a class of enzymes that have emerged as potential targets for CIPN-preventing drugs. An increase in MMP2 and MMP9 and a decrease in metallopeptidase inhibitor 1 (TIMP1), a strong endogenous MMP9 inhibitor, were observed in a paclitaxel mouse model of CIPN.

Intrathecal injections of exogenous TIMP1 or a monoclonal antibody targeting MMP9 (MMP9 mAb) significantly prevented and reversed paclitaxel-induced mechanical allodynia in mice. This antibody significantly decreased oxidative stress and levels of neuroinflammatory IL-6 and TNFα. MMP9 mAb prevented paclitaxel-induced intraepidermal nerve fiber loss. Hence, it was suggested that MMP9 mAb has therapeutic potential for the treatment of CIPN. Importantly, a humanized MMP9 antibody (andecaliximab) has reached advanced clinical trials for the treatment of colitis (ClinicalTrials.gov: NCT02405442) [139, 140] and alimentary tract cancer (ClinicalTrials.gov: NCT01803282) [141, 142]; hence, it may also be repurposed for CIPN [143].

Our unpublished studies, as well as research results from other laboratories, indicate that minocycline, a tetracycline antibiotic, a microglia inhibitor and a MMP9 blocker, alleviates the development and symptoms of CIPN caused by oxaliplatin, cisplatin, paclitaxel and bortezomib [144–148].

Animal models have also indicated that the targeting of receptors expressed within pain pathways might be an important pharmacological approach for CIPN-preventing drugs. In mice, the blockade of muscarinic receptors with the use of benztropine prevented both acute and chronic symptoms of peripheral neuropathy caused by oxaliplatin. In in vitro studies, benztropine also attenuated electrophysiological alterations due to CIPN and prevented the decrease in neuronal density in the paws of mice that were injected with oxaliplatin. A lower tumor growth was observed in mice receiving benztropine when compared to untreated animals, and benztropine acted synergistically with oxaliplatin by increasing its anticancer effect. This activity was attributed to the influence of benztropine on ROS imbalance in tumor cells [149].

Nicotine is under investigation as another potential therapeutic strategy within the cholinergic system for both the prevention and the treatment of paclitaxel-induced CIPN. Available data suggest that the targeting of the nicotinic-acetylcholine-receptor-mediated pathway may be a promising strategy for the prevention and treatment of CIPN-induced symptoms, in particular mechanical allodynia and intraepidermal nerve fiber loss [150]. A primary concern is that nicotine may also stimulate tumor growth, and smoking history has been classified as a risk factor for CIPN. The research is focused on nicotinic receptor agonists as novel strategies for the prevention and treatment of CIPN [150, 151].

R-47, an α7 nAChR silent agonist, showed promising activity for the prevention and treatment of CIPN caused by paclitaxel. Its activity did not interfere with the antitumor activity of paclitaxel. No tolerance developed following repeated administration of R-47, and R-47 lacked intrinsic rewarding effects. Additionally, R-47 did not influence the rewarding effects of nicotine in the conditioned place preference test in mice, and it did not enhance mecamylamine-precipitated withdrawal [151].

Cebranopadol (a.k.a. GRT-6005) is a first-in-class potent analgesic compound with agonistic activity at nociceptin/orphanin FQ and opioid receptors [152]. It has been recently developed in phase II clinical trials for the treatment of painful diabetic neuropathy or cancer pain. Subcutaneously administered cebranopadol reduced cold allodynia in oxaliplatin-treated mice, and this effect was observed both in the acute and the late phase of CIPN [153].

In rodent models, the administration of carvedilol, a β-adrenolytic drug with cardioprotective properties, reduced levels of nitrotyrosine and subsequently increased the expression of mitochondrial SOD in both sciatic nerves and dorsal root ganglia. Based on this mechanism of action, it was suggested that this drug might have the potential for preventing an alteration in mitochondrial membrane potential in nerves and the loss of intraepidermal nerve fiber density. Several ongoing studies evaluating carvedilol for a variety of clinical conditions, but, at present, there is no clinical study for the prevention of CIPN [4].

A series of novel amide derivatives of 1,3-dimethyl-2,6-dioxopurin-7-yl-alkylcarboxylic acids, analogs of a TRPA1 antagonist, i.e., HC-030031, were assessed in vitro and in vivo. These compounds were identified as dual TRPA1 channel antagonists and phosphodiesterase 4B/7A inhibitors. They reduced tactile allodynia in oxaliplatin-treated mice and showed TNFα-lowering properties; therefore, they are regarded as interesting drug candidates for CIPN [154].

Hyperpolarization-activated cyclic nucleotide-gated (HCN) channels are overexpressed in various inflammatory and neuropathic pain states, and a novel HCN1 inhibitor, MEL57A, has recently demonstrated antiallodynic and antihyperalgesic properties in oxaliplatin-treated rats. HCN1 channels were proposed as a new drug target in CIPN, and HCN1-selective inhibitors might be a new class of medications for CIPN [155].

Many mechanisms underlying CIPN overlap and can reinforce each other. Thus, a combination drug therapy might also be an interesting and more efficacious (compared to monotherapy) approach to CIPN prevention [120]. Although there are no clear algorithms for the clinical use of combined drugs in CIPN patients, data from preclinical research indicate that certain drug combinations might effectively attenuate symptoms of CIPN [156]. In our recent study, a hyperadditive effect of combined subanalgesic doses of ambroxol and pregabalin on cold allodynia was demonstrated in oxaliplatin-treated mice. This effect was particularly strong when both drugs were given simultaneously or sequentially, i.e., 4 h apart. These results point out a synergistic reaction between Na_v_ and Ca_v_ channel inhibitors in CIPN caused by oxaliplatin [156].

Mesenchymal stem cell (MSC) therapy is also an emerging attractive approach for CIPN. Evidence has demonstrated that MSCs reduce oxidative stress, neuroinflammation, and neuronal cell apoptosis and stimulate axon regeneration after nerve damage. Hence, MSC-based therapies may provide a new therapeutic strategy for patients suffering from CIPN. Combined therapy using ‘traditional’ pharmacological agents and MSCs might also be an interesting approach for CIPN prevention and treatment [157].

Several studies have also been conducted to assess whether targeting pathways associated with gut microbiota might be a way to overcome CIPN [158]. Probiotics show therapeutic effects in various diseases, such as travelers’ diarrhea or irritable bowel syndrome, and previous studies have also confirmed their protective role in various nociceptive pain states [159–161], but, at present, little is known about their potential effect on CIPN.

Using F11 cells, the probiotic DSF was tested for its ability to attenuate paclitaxel-induced neuropathic pain. The results obtained suggested its potential use as an adjuvant agent for CIPN [162]. The following mechanisms explain a beneficial effect of probiotics through which they might inhibit CIPN: a reduction in proinflammatory cytokine levels, the regulation of the balance between anti-inflammatory and proinflammatory cytokines, the inhibition of neutrophil infiltration and increased intestinal permeability, augmented production of short-chain fatty acids, the inhibition of ammonia formation, changes in the expression levels of toll-like receptors: TLR2 and TLR4, the maintenance of mucosal integrity through mucin secretion and the activation of suppressive T-cell populations [158]. Here, it should be strongly emphasized that the research on the gut microbiome in CIPN is still at an exploratory stage, and potential therapeutic methods based on microbiota manipulation require further preclinical and clinical research. In particular, clinical trials assessing the use of probiotics to prevent or treat CIPN are a strong medical demand [158].

The efficacy of complementary therapies in the prevention and treatment of CIPN has also been a subject of research. Herbal medicinal therapies (e.g., *Acorus calamus*, *Cannabis* species, *Matricaria chamomilla*, *Ginkgo biloba*, *Curcuma longa*, *Camellia sinensis*) counteract phenomena underlying CIPN, i.e., they reduce oxidative stress and attenuate inflammation in animals [163], but their utility for CIPN prevention requires further investigation to confirm their efficacy and safety [164–167].

In addition to novel pharmacological targets for the prevention and/or treatment of CIPN, nonpharmacological approaches were also assessed. Interestingly, a growing body of literature suggests that exercise can prevent CIPN. In rodent models of CIPN, volitional wheel running reduced both the development and the maintenance of cold and mechanical allodynia. This finding was confirmed in patients assigned to the exercise group during chemotherapy who reported less severe thermal and sensory symptoms of CIPN than patients who received chemotherapy alone [94].

Preclinical data regarding potential future preventative therapies for CIPN are summarized in Table 3.

**Table 3:** Potential future preventative therapies for CIPN

Target	Compound/drug candidate	Mechanism underlying CIPN prevention	References
SARM-1	Small-molecule SARM-1 inhibitors, NAD1 precursors	Axonal degeneration pathway inhibition	[114]
Hsp90	Ethoxyquin	Modulation of the chaperone activity of Hsp90—axonal degeneration prevention	[117, 118]
Bclw	Bclw mimetics	Increase in Bclw level—axonal degeneration inhibition	[101, 119]
ROS	Pifithrin-µ, histone deacetylase 6 inhibitors, Metformin, peroxynitrite decomposition catalysts, IL-10, niclosamide MSCs Herbal medicinal therapies	Mitochondrial oxidative stress reduction—cell death prevention	[122–124] [124] [44, 74, 125] [126] [125, 127] [129] [157] [163–167]
TNFα	Etanercept	Reduction of neuroinflammation	[128]
Chemokines and chemokine receptors: IL-8 and CXCR1/CXCR2 receptors CX3CL1 (fractalkine) and CX3CR1 receptors	Reparixin, DF2726A Gastrodin	IL8-CXCR1/CXCR2 system inhibition—suppression of immune responses CX3CL1-CX3CR1 system inhibition—spinal microglia inhibition	[131] [132, 133] [134–136]
MMPs	TIMP1, andecaliximab Minocycline	MMPs inhibition—reduction of neuroinflammation and decreased intraepidermal nerve fiber loss	[139, 143] [144–148]
Cholinergic M receptors	Benztropine	M receptor antagonism—neuronal density loss prevention	[149]
Cholinergic N receptors	R-47	N receptor stimulation—Intraepidermal nerve fiber loss prevention	[150, 151]
NOP receptors	Cebranopadol	NOP agonism—cold allodynia/hyperalgesia prevention	[153]
Adrenergic β receptors	Carvedilol	β receptor antagonism and ROS inhibition—intraepidermal nerve fiber loss prevention	[4]
TRPA1 channels	HC-030031, and analogs	TRPA1 antagonism—tactile allodynia and cold allodynia/hyperalgesia reduction	[154]
Na_v_ channels	Ambroxol	Na_v_ channel inhibition—decreased neuronal cell excitability	[156]

*SARM-1* sterile alpha and TIR motif-containing protein 1, *NAD1* NADH dehydrogenase subunit I, *Hsp90* heat shock protein 90, *ROS* reactive oxygen species, *IL* interleukin, *MSCs* mesenchymal stem cells, *TRPA1* transient receptor potential ankyrin-repeat 1 channel, *CXCR1/CXCR2* IL-8 (CXCL8) CXCR1 and CXCR2 receptors, *CX3CR1* CX3CL1 (fractalkine) receptor, *MMPs* matrix metalloproteinases, *NOP* nociceptin opioid peptide receptor, *Na*_*v*_ voltage-gated sodium channel

## Conclusions

Although preclinical models reflecting CIPN have provided a deeper insight into mechanisms that initiate and maintain antitumor drug-induced neurotoxicity, this clinical entity currently cannot be prevented. New therapeutic approaches and drug targets should be assessed, and improved clinical trials need to be designed to search for drugs for CIPN prevention. This research should address genotypic profiles of patients suffering from CIPN, and the knowledge regarding genetic CIPN susceptibility needs to be incorporated into clinical trials. This research not only would contribute to recent advances in basic and translational research for CIPN prevention [168] but also would enable the prediction of patients’ susceptibility to CIPN development prior to starting chemotherapy based on CIPN-inducing drugs. In addition, pharmacogenomics studies could be useful in the identification of mutations in genes influencing antitumor drug metabolism, and this could also be an important source of data for optimal dosing of these drugs in patients [101].
